# Supplementary material for: Implementing a smoking cessation intervention for people experiencing homelessness: Participants’ preferences, feedback, and satisfaction with the ‘power to quit’ program
Source: PLoS One. 2022 Jun 24;17(6):e0268653. doi: 10.1371/journal.pone.0268653 (PMC9231781; doi:10.1371/journal.pone.0268653)
Supplement: Attachment — Submitted filename: Response to reviewers.docx [file pone.0268653.s001.docx]

Professor Joerg Heber
Editor-in-Chief
PLOS ONE

26^th^ November 2021

PONE-D-21-22291

Implementing a smoking cessation intervention for homeless smokers: Participants preferences, feedback, and satisfaction with the ‘power to quit’ programme

We would like to thank the academic editor and the reviewers for their constructive feedback and helpful comments on our manuscript titled ‘Implementing a smoking cessation intervention for homeless smokers: Participants preferences, feedback, and satisfaction   with the ‘power to quit’ programme’. Please see below our responses.

**JOURNAL REQUIREMENTS**:

**Comment 1.** Please ensure that your manuscript meets PLOS ONE's style requirements, including those for file naming.

**Our Response (1):** This manuscript meets PLOS ONE's style requirements

**Comment 2**. Please ensure you have included the registration number for the clinical trial referenced in the manuscript.

**Our Response (2):** Trial Registration number has been stated (Line 111)

**Comment 3.** We note that you have stated that you will provide repository information for your data at acceptance. Should your manuscript be accepted for publication, we will hold it until you provide the relevant accession numbers or DOIs necessary to access your data. If you wish to make changes to your Data Availability statement, please describe these changes in your cover letter and we will update your Data Availability statement to reflect the information you provide.

Our Response (3): Data will be made available on request. This is now being included in the cover letter.

**Comment 4.** Please include your full ethics statement in the ‘Methods’ section of your manuscript file. In your statement, please include the full name of the IRB or ethics committee who approved or waived your study, as well as whether or not you obtained informed written or verbal consent. If consent was waived for your study, please include this information in your statement as well.

**Our Response (4):** Full name of the IRB as well as statement on informed consent has been stated. Study procedures were approved and monitored by the Institutional Review Board of the University of Minnesota Medical School (Study Number: 1307M39761). Written informed consent was obtained from participants prior to data collection. (Lines 306 - 308)

**REVIEWER 1**

**Comment 1a:**  Currently, there is little description of the survey in the methods section. How were participants invited to take part.

**Our Response (1a):** ‘Researchers recruited participants by conducting health fairs, holding informational interviews, posting flyers, and announcing the study at homeless shelters. Participants also helped to recruit by word of mouth.’ This has been stated in the methods section (Line 148-150) The eligibility criteria is also stated (Lines 150 – 167)

**Comment 1b:** What was the method of administration/data collection,

**Our Response (1b):** Validated questionnaires were used to collect survey data. Metric measurements of height and weight were collected to calculate body mass index. Carbon monoxide (CO) and saliva cotinine was assessed as biomarkers of tobacco use. The participants were asked to exhale into a carbon monoxide monitor. Patch adherence was measured by: ‘patch checks’ (visual verification of whether a participant was wearing a patch); ‘patch counts’ (documenting the number of patches left in the participant’s possession); and administration of the Morisky scale, a self-reported adherence scale modified to assess adherence to NRT patch

Detailed method of data collection has been stated in the methods (Lines 170 – 196)

**Comment 1c:** was there a participant incentive for completing the survey, how long was the survey?

**Our Response (1c):** Incentives were given as compensation for participants time and effort. At each of the 15 visits, participants received incentives. For attending the final 6-month visit, participants received a $40 gift card and a sweatshirt. For participants who attended all 15 sessions, the monetary incentives totaled $275 over 6 months. The study lasted for 26 months. (Details in Lines 131-140)

**Comment 1d:** The methods would also benefit from a full description of what the survey covered. The results suggest that more was asked than is currently outlined in the methods.

**Our Response (1d):** The methods section has been improved upon and contains information on:

An overview of the study, aim of the study and the intervention components (Line 111-140)

Recruiting of participants and eligibility criteria ( Lines 148 -167)

Data collection (Lines 170 -196)

**Comment 2:** Was all the data used in the paper collected at the 26-week survey? It would be useful to clarify this, i.e. was the demographic and other data collected at 26-weeks or was this collected earlier in the trial? This is important to highlight because some of the smoking data, if collected at 26 weeks, will likely have been influenced by participation in the trial, for example the % reporting an unsuccessful quit attempt in the last year and the high confidence to quit score could have been a direct result of the trial, and not therefore suggestive of homeless populations, and should be noted.

Our Response (2): Study data was collected at baseline, week 1, week 2, week 4, week 6, week 8, week 10, week 12, week 14, week 16, week 18, week 20, week 24, and week 26. However, data analyzed in this study was limited to the baseline and week 26 feedback survey. The detailed information is provided in the Data collection section (Lines 179-196)

**Comment 3:** How was the survey (and importantly, the questions) developed? Were they tested for participants’ understanding and relevance? There are potential issues with some of the main questions asked. For example, the question, ‘Which items were most helpful?’ This question is open to interpretation. Did the authors mean which items were most helpful for reducing smoking or quitting? The inclusion of ‘reading materials’ or ‘community mobile contacts’ suggest that the question could also have been interpreted as ‘most helpful sources of information?’

Our Response (3):The survey tool was developed by the research team with the aim of assessing participants satisfaction with the research program and its components. Prior to the start of the survey, all questions were pretested for understanding and relevance. The survey was administered by trained interviewers to avoid possible misinterpretations of the questions.

**Comment 4:** The monetary/voucher incentives were particularly valued by homeless study participants. This is unsurprising, although they were not linked with programme satisfaction. The authors write that “incentives or specific components of the programme may not have significantly affected participant motivation to participate in and complete the study”. What were the incentives in the study used for, e.g. enrolment, attending follow-up appointments, motivation for smoking abstinence? This needs to be clarified as currently the paper doesn’t tell us very much about how incentives were used in the trial, and therefore the implications for future interventions are limited.

**Our Response (4):** Incentives were given as compensation for participants time and effort. Incentives were provided at each of the 15 visits. They were provided when participants completed surveys, attended brief retention visits, attended treatment visits, and at the follow-up 6 month visit. (Details in Lines 131-140)

**REVIEWER 2**

**Comment 1:** I might consider changing the language to reflect people or populations experiencing homelessness or people experiencing homelessness who smoke as an indicator that this population is constantly changing and is dynamic based on economic and other circumstances, and move away from calling the population, "the homeless" or "homeless smokers" which signifies a static condition and potentially a character trait (which we know it is not). This might be viewed as less stigmatizing. I would suggest making this change throughout the manuscript.

**Our Response (1):** The language has been changed to reflect populations experiencing homelessness or people experiencing homelessness.

**Comment 2:** In the abstract, I might include how satisfaction was qualitatively assessed. I might bring in the point that the rate of satisfaction was high among African American participants in part because there may have been race concordance between study staff, community advisory board and AA participants. I think that is the salient point here that it is helpful to have staff that have lived experiences of participant in the study to improve the effectiveness of the study.

**Our Response(2):** The assessment of satisfaction has been included in the abstract (Line 32).

Race concordance influencing satisfaction has been included in the abstract (Lines 49 – 52)

Race concordance influencing satisfaction has been included in the discussion ab-initio (Line 415 – 429)

**Comment 3** Methods: I might include where the study sites were in Minneapolis and St. Paul - I recall they were in shelters in these cities. Did participants have to commute? What about timings for the assessment and counseling sessions -- were they flexible?

**Our Response(3):** Study sites were situated within homeless shelters and/or homeless service centers that are located in the downtown/city center easily accessible by public transportation such as city-operated buses and light rail. (Line 146 – 148)

Because of the nature of the study population, timings for assessments and counselling sessions were flexible. (Line 180 – 181)

**Comment 4:** The authors alluded to this but was the validity of the satisfaction question in this sample -- could the authors conduct an internal validity of this question in this sample?

**Our Response(4):** The importance of satisfaction is widely recognized in substance use treatment therefore future interventions should aim to assess the internal validity of this specific construct.(Line 484-489) Unfortunately, the Power to Quit study concluded in 2011 therefore we are unable to assess the internal validity of this question within our study sample. In addition, internal validation of the satisfaction question was not planned a priori therefore the satisfaction questions were not constructed in a way that would allow us to go back and run statistical analysis such as confirmatory factor analysis.

**Comment 5:** Page 7, Line 150 -- which item was most helpful to you -- is this in relation to smoking cessation or general life?

**Our Response(5):** We thank the reviewers for this comment. The question items that asked participants to state the items they found most helpful was with respect to smoking cessation. The survey was administered by trained interviewers who were able to provide additional context to questions where needed. The respondents were aware that the questions asked were with respect to smoking cessation.

**Comment 6:** The authors described many covariates, e.g., health and psychosocial covariates, smoking-related covariates, substance use and dependence covariates -- while these are interesting, I wonder how much is relevant to the analysis on satisfaction as none of these variables are controlled for in the model. I might consider taking these out of this paper and referring to the original Power to Quit study findings for descriptive statistics. It does not seem that satisfaction differed based on mental health or substance use based on these findings and if so, might clarify and explain why that might be in the results and discussion.

**Our Response(6):** We included information on the covariates that were used in the analysis of this paper. We have expunged information other covariates (self-efficacy to refrain from smoking) that were not included in the descriptive and bivariate analysis.

In multivariable analysis, variables significant at p<0.10 in the bivariate analysis were imputed in the logistic regression (Line 298-299 ) We feel it is important to show that some of the covariates did not show statistically significant association with satisfaction. Hence, we have included table 4 showing the bivariate analysis (Line 395).

**Comment 7a:** On the results/discussion, I might consider discussing in the results, how satisfaction varied for participants who were abstinent at 6 months vs. not; for participants in the intervention vs control (I did not see this distinction and it seems relevant)

**Our Response(7a):** The number of participants who successfully quit at the end of the survey was very small. As a result, such statistical analysis would not have yielded any meaningful statistically significant differences. We have highlighted this as a study limitation and highlighted it as an area for future research.(Lines 476-484)

**Comment 7b:** On the results/discussion, one of the participants' feedback was on continuing patch and counseling if that option was available to participants -- and it has implications for extended duration interventions for PEH, perhaps it might be good to discuss this point in the discussion.

**Our Response(7b):** Thank you for this recommendation. We have updated our results and discussion sections to include this discussion point. (Table 2, Line 489 – 500)

**Comment 7c:** On the results/discussion - when I read that African American/Black participants had higher satisfaction levels, I immediately thought this was because staff might have been African American/Black. Wonder if there was a way to create a variable on racially concordant staff/participant dyad/group variable vs. not and see if that is associated with satisfaction. I think this finding is really important for anyone doing work among PEH, as African American/Black folks are over-represented in populations experiencing homelessness in large urban cities, and calls for having study staff/teams that are representative of the lived experiences and gender/racial.ethnic diversity of PEH.

**Our Response(7c):** Thank you for this comment. While we agree that this would have enriched the study findings, we did not set out to assess this relationship in the initial study. As a result, we are unable to generate a variable for racially concordant staff/participant dyad/group and include this in our analysis. However, we have included this point as an area for future research.

(Line 472 – 474 )

**Comment 7d:** Can the authors comment on when should satisfaction be ascertained (e.g., at what time points -- all study visits), and how can intervention design be malleable to changing views on satisfaction during an intervention among PEH.

**Our Response(7d):** Satisfaction should be at the least assessed at the end of the study. The pros and cons of assessing satisfaction at multiple time points should be balanced. The pro of assessing satisfaction is the ability to obtain more information that will help to inform future studies. This would be appropriate in a study with a pragmatic design where design changes are built apriori on the study protocol. However, repeated measures of satisfaction may have unintended consequences. Participants may be more prone to giving socially desirable responses, the repeated surveys may become too burdensome for participants and may negatively impact attrition, and the repeated measures may become too burdensome for the research team. Furthermore, changing views on satisfaction should be incorporated into the study without changing randomization of the participants and without creating barriers to carrying out the study.

Yours sincerely,

Oluwakemi Ololade Odukoya
On behalf of the study investigators
