# Supplementary material for: Implementing a smoking cessation intervention for people experiencing homelessness: Participants’ preferences, feedback, and satisfaction with the ‘power to quit’ program
Source: PLoS One. 2022 Jun 24;17(6):e0268653. doi: 10.1371/journal.pone.0268653 (PMC9231781; doi:10.1371/journal.pone.0268653)
Supplement: Attachment — Submitted filename: Response to reviewers (2).docx [file pone.0268653.s002.docx]

Professor Joerg Heber
Editor-in-Chief
PLOS ONE

25^th^ March 2022

PONE-D-21-22291

Implementing a smoking cessation intervention for homeless smokers: Participants preferences, feedback, and satisfaction with the ‘power to quit’ programme

We would like to thank the academic editor and reviewer for their constructive feedback and helpful comments on our manuscript titled ‘Implementing a smoking cessation intervention for homeless smokers: Participants preferences, feedback, and satisfaction   with the ‘power to quit’ programme’. Please see below our responses.

**EDITORS COMMENTS**:

**Comment 1.** First, the Data Availability field states that Yes all data are fully available without restriction, but this does not appear to be the case per the cover letter. If data are only available on request, please state why there are legal or ethical restrictions on sharing data. The doi in dryad does not lead to public data set. Please review the PLOS ONE FAQ regarding data sharing: https://journals.plos.org/plosone/s/data-availability

**Our Response (1):** All data are fully available without restriction an can be accessed at <https://www.openicpsr.org/openicpsr/project/165321/version/V1/view> This has also been stated in the cover letter

**Comment 2**. Line 58: 20% is outdated if referring to U.S, which is now around 14%.

**Our Response (2):** The information on the prevalence of smoking in the general population in the US has been updated (Line 59). The reference has also been updated (Line 520-522).

**Comment 3.** Line 292-293: Please rephrase to clarify that participants had a positive screen for alcohol or drug dependence rather than identifying them as alcohol/drug dependent (given the relationship between screeners and actual diagnostic status of substance use disorder is far from 100%). Same guidance for Table 1.

Our Response (3): Thank you for your comment. The term “Alcohol/drug dependent” has been re-phrased as “had a positive screen for alcohol or drug dependence” (Line 297-298, Table 1 Line 307, Table 4 Line 369 )

**Comment 4.** Table 1: Please relabel rows to 1, 2, >3. It seems odd and minimizing to use the word “just” as a qualifier for the number of times experiencing homelessness.

**Our Response (4):** The rows have been re-labelled as Once, Twice, Thrice (Table 1 Line 307, Table 4 Line 369 )

**Comment 5:** Table 3: Overall program satisfaction – please report frequency/percentage for all 5 response options.

**Our Response (5):** The frequency/percentage values have been reported for all 5 responses. (Table 3 Line 362)

**Comment 6:** Table 4: In table note please remind the reader of how ‘satisfied’ and ‘not satisfied’ were coded. Why was ‘somewhat satisfied’ coded as ‘not satisfied’? I can see a justification for combining response options 1-3, but ‘somewhat satisfied’ doesn’t seem like it fits under the umbrella of ‘not satisfied’. I won't require to re-code and rerun all analyses, but please provide a justification for this decision if you prefer to maintain this coding scheme, but adjust labels (e.g., ‘Very satisfied vs No very satisfied’.

**Our Response (6): The options for satisfaction were** 1)Not satisfied at all; 2) Somewhat unsatisfied; 3) Neutral; 4) Somewhat satisfied; 5) Very satisfied. There was no option stated as “somewhat satisfied” rather this was “somewhat unsatisfied” We believe that the options Not satisfied at all and somewhat unsatisfied both reflect some form of dissatisfaction, hence they were categorized together as “Not very satisfied” We have reminded the readers of how overall program satisfaction was re-coded. The labels have been adjusted as ‘Very satisfied vs Not very satisfied’ (Table 4 Line 369, Line 373-375)

**Comment 7:** Table 4: Please justify your alpha of 0.10 for Table 4.

**Our Response (7):** In multivariable analysis, variables significant at p<0.10 in the bivariate analysis were imputed in the logistic regression in order to include variables tending towards a positive significance. (Line 271-272)

**Comment 8:** Table 5: Could include the 95% CI in parentheses after the AOR (e.g., 1.005 (0.977, 1.035)). Please label as 95% CI in table note or column header (per Reviewer 3).

**Our Response (8):** The 95% CI is stated in parentheses after the AOR. The column header is labeled as **AOR(95% CI Lower limit , Upper limit) (Table 5 Line 393)**

**Comment 9:** Reviewer 3 provides useful suggestions that I recommend you incorporate into the revised manuscript. Given that this manuscript has already been reviewed by two peer reviewers, I would not require you to make the following changes recommended by Reviewer 3:
-Participants (line 150): readers can refer to your citation of the main design/outcomes papers
-Data Collection (Line 176-178): you already state only baseline/26wk are presented here.
-Table 1: do not need to recode age. Cosmetic changes to tables will occur during copy-editing, not necessary at this stage.

**Our Response (9):** Comments well taken

**REVIEWER 3**

**Comment 1:**  Exposure variables
Line 195, coding labelling to be fully provided e.g. 1(most helpful), 2( ), 3(not helpful). Likewise Line 201 e.g. 1(liked the most), 2( ), 3(did not like it)

**Our Response (1):** Thank you for your comment. This has been done. (Line 197, Line 203-204)

**Comment 2:**  Line 264, Analysis to be written as Statistical analyses.

**Our Response (2):** Thank you for your comment. This has been done. (Line 266)

**Comment 3:**  Line 269, for the statement ‘In addition, we controlled for study arm,’ the reason to analyze as combined groups and not groups comparison to be clearly stated before displaying the results.

**Our Response (3):**We thank the reviewer for this comment. Study arm was not controlled for in the regression model. This has been corrected in the methods (Statistical analysis) (Line 272)

**Comment 4:**  Results
Line 284, full name for GED to be provided.

**Our Response (4):** This has been done (Line 288-289).

**Comment 5:**  Line 322. 21% to be replaced with 21.0%.

**Our Response (5):** This has been done (Line 327)

**Comment 6:**  Line 324, Over 80% to replaced with 81.6%

**Our Response (6):** This has been done (Line 329)

**Comment 7:**  Line 337, typo ‘thatthe’

**Our Response (7):** This has been done (Line 343)


**Comment 8:**  Line 303 Table 1, title too brief. For monthly income, the symbol for the income category is incorrect (different to Line 214). For the homelessness characteristics ‘just once, One to three times(twice) and More than three times (≥3)’ are confusing Perhaps just state once, twice, thrice or more. Drug dependent and not drug dependent to be unbold.

**Our Response (8):** This has been done (Table 1 Line 307 - 308)

**Comment 9:**  Line 303 Table 1 and Line 330 Table 2, all the data to be presented (i.e No to be included) while missing data to be denoted in the table footnote and n to be stated for all variables.

**Our Response (9):** All the data has been presented. However, missing data was not shown in table, rather ‘n’ was stated for each variable. (Table 1 Line 308, Table 2 Line 336)

**Comment 10:**  Line 356 Table 3, the figure 4 or 5 in the subcategory to be spelled out or denoted in the table footnote. n to be stated and any missing data to be denoted.

**Our Response (10):** Thank you for this comment. This has been effected (Table 3 Line 362).

**Comment 11:**  Line 360 Table 4, n to be stated for each variable. Actual symbol chi-square X^2 to be used. The chi square value to be reduced to 2 decimal points and the decimal points for p value to be standardized. All the statistical tests used in Table 4 to be denoted in the table footnote. For the age, depression, stress, age started smoking regularly, number of 24 hour quit attempts past year, confidence to quit variables, the data were presented as mean± sd. The statistical test to be stated. If chi-square test was employed, the categories of each variable and frequency to be displayed. For the self-reported general health category poor, 14.2% to be replaced with 14.3%. Please re check the chi-square value and p value for the variable ‘top ranked incentive’.

**Our Response (11):** The comments have been effected (Table 4 Line 370)

**Comment 12:**  The word p value or p-value to be consistent with Table 5. # was mentioned in table footnote but the label nowhere found in the table.
**Our Response (12):** This has been done (Table 5 Line 393)

**Comment 13:**  Line 373 Table 5, the model summary such as pseudo R^2 and goodness of fit test to be provided. 95%CI to be stated before lower and upper limit are stated.

**Our Response (13):** pseudo R^2 and goodness of fit test have been provided (Table 5 Line 393)

**Comment 14:**  Line 367, the sentence ‘Race was with a predictor of overall programme satisfaction.’ requires revision.
**Our Response (14):** This has been corrected (Line 382)

**Comment 15:**  Line 376, p=.05 to be replaced with p < 0.05.

**Our Response (15):** This has been corrected (Line 398)

**Comment 16:**  Discussion
Line 427, 435 & Line 437, typos thesurvey, Fourth,, Fifth..

**Our Response (16):** This has been corrected (Line 447, 455, 457)

**Comment 17:** Was there any other possible bias arising from the interviewing process?

**Our Response (17):** There is also a possibility of non -response bias which arises when respondents are systematically different from non-respondents. Prior to data collection, measures were instituted to limit such biases and this has been included in the text. (Line 455-460).

Yours sincerely,

Oluwakemi Ololade Odukoya
On behalf of the study investigators
